# Low-Dose Cd Induces Hepatic Gene Hypermethylation, along with the Persistent Reduction of Cell Death and Increase of Cell Proliferation in Rats and Mice

**DOI:** 10.1371/journal.pone.0033853

**Published:** 2012-03-23

**Authors:** Bo Wang, Yang Li, Yi Tan, Xiao Miao, Xu-Dong Liu, Chen Shao, Xiao-Hui Yang, Subat Turdi, Li-Jie Ma, Jun Ren, Lu Cai

**Affiliations:** 1 Department of Pathophysiology, Prostate Diseases Prevention and Treatment Research Center, Norman Bethune College of Medicine, Jilin University, Changchun, Jilin, China; 2 Department of Pathology, Inner Mongolia Forestry General Hospital, Yakeshi, Inner Mongolia, China; 3 Department of Pediatrics, The University of Louisville, Louisville, Kentucky, United States of America; 4 Department of Ophthalmology, The Second Hospital of Jilin University, Changchun, Jilin, China; 5 Department of Emergency, Affiliated Hospital, Inner Mongolia Medical College, Hohhot, Inner Mongolia, China; 6 Center for Cardiovascular Research and Alternative Medicine, University of Wyoming, Laramie, Wyoming, United States of America; 7 Department of Pharmacology, Inner Mongolia Medical College, Hohhot, Inner Mongolia, China; 8 Departments of Radiation Oncology, Pharmacology and Toxicology, the University of Louisville, Louisville, Kentucky, United States of America; University of Hong Kong, China

## Abstract

**Background:**

Cadmium (Cd) is classified as a human carcinogen probably associated with epigenetic changes. DNA methylation is one of epigenetic mechanisms by which cells control gene expression. Therefore, the present study genome-widely screened the methylation-altered genes in the liver of rats previously exposed to low-dose Cd.

**Methodology Principal Findings:**

Rats were exposed to Cd at 20 nmol/kg every other day for 4 weeks and gene methylation was analyzed at the 48^th^ week with methylated DNA immunoprecipitation-CpG island microarray. Among the 1629 altered genes, there were 675 genes whose promoter CpG islands (CGIs) were hypermethylated, 899 genes whose promoter CGIs were hypomethylated, and 55 genes whose promoter CGIs were mixed with hyper- and hypo-methylation. Caspase-8 gene promoter CGIs and TNF gene promoter CGIs were hypermethylated and hypomethylated, respectively, along with a low apoptosis rate in Cd-treated rat livers. To link the aberrant methylation of caspase-8 and TNF genes to the low apoptosis induced by low-dose Cd, mice were given chronic exposure to low-dose Cd with and without methylation inhibitor (5-aza-2′-deoxyctidene, 5-aza). At the 48^th^ week after Cd exposure, livers from Cd-treated mice displayed the increased caspase-8 CGI methylation and decreased caspase-8 protein expression, along with significant increases in cell proliferation and overexpression of TGF-β1 and cytokeratin 8/18 (the latter is a new marker of mouse liver preneoplastic lesions), all which were prevented by 5-aza treatment.

**Conclusion/Significance:**

These results suggest that Cd-induced global gene hypermethylation, most likely caspase-8 gene promoter hypermethylation that down-regulated its expression, leading to the decreased hepatic apoptosis and increased preneoplastic lesions.

## Introduction

Cadmium (Cd) is a nonessential metal responsible for several human diseases and has been classified as a human carcinogen by the National Toxicology Program of USA. Waisberg and colleagues proposed multiple mechanisms for Cd-associated carcinogenesis, including modulation of gene expression and signal transduction, interference with enzymes from the cellular antioxidant system and generation of reactive oxygen species, inhibition of DNA repair, increase in DNA methylation, induction of apoptosis, and disruption of E-cadherin-mediated cell-cell adhesion [1]. Among these possible mechanisms induction of aberrant DNA methylation may be predominant in Cd carcinogenesis at the molecular level.

An epigenetic mechanism of proto-oncogene gene activation by Cd involves inhibition of DNA methylation, a cellular tool for the regulation of gene repression. Hypomethylation has been reported to be associated with overexpression of proto-oncogenes essential for carcinogenesis [2]. In the mammalian genome, DNA methylation is one of the most commonly occurring epigenetic events resulting in the covalent addition of a methyl group at the carbon 5 position of the cytosine ring. Cytosine methylation is a chemically stable mark that may establish, or follow as a consequence of, the packaging of a particular region into silent chromatin. Therefore, identification of aberrant genomic DNA methylation that is associated with carcinogenesis should identify targets that are important for disease progression [3]#.

DNA hypermethylation in cancers was observed very often in CpG islands (CGIs) in gene promoters, resulting in down-regulation of tumor suppressor genes [3]. Both promoter methylation-mediated silencing of tumor suppressor genes and activation of oncogenes resulted from promoter hypomethylation have been well documented in various tumor models. For instance, aberrant cytosine methylation patterns in CGIs are hallmarks of human cancers, and the methylation of CGIs in gene promoters acts as a powerful suppressor of transcriptional activity [3]. Specifically DNA hypermethylation is a well described phenomenon in common hepatocellular carcinoma (HCC) [4] and also in hepatocellular adenoma [5].

Caspase-8 (CASP8), a cysteine protease, involves in both death-receptor-dependent and -independent manners during apoptosis. CASP8 functional loss was previously attributed to DNA hypermethylation in as many as 61% of human neuroblastoma cases [6]. In addition, the promoter CGIs of the CASP8 gene were fully methylated in all liver tissues from patients with HCC tested whereas unmethylated in all four normal liver tissues from healthy donors. This suggests that hypermethylation of CASP8 may contribute exclusively to HCC via its inactivation in regulating apoptotic cell death [4].

*In vitro* studies on carcinogenesis indicated that Cd exposure malignantly transforms various human and rodent cells, which give rise to aggressive cancers when injected into mice [7], [8]. DNA methylation has been proved in the Cd-exposed cells *in vitro* [2], [9]. Therefore, the aberrant methylation changes may be the potential mechanisms for Cd-induced malignant transformation and eventual carcinogenesis. To date, however, no evidence is available with regards to the epigenetic effect of chronic exposure of low-dose Cd in an *in vivo* model. Recently we noted a persistent increase of cell proliferation in the liver of rats at the 48^th^ week after exposure to low-dose Cd for 4 weeks [10]#.

Therefore, the present study was designed to further examine whether chronic exposure to low-dose Cd causes aberrant DNA methylation and if so, whether these late effects of previous Cd exposure are associated with the aberrant DNA methylation in rats and mice models.

## Results

### Livers of Rats Previously Exposed to Chronic Low-Dose Cd Showed a Low Incidence of Apoptotic Cell Death and Increased DNA Double Strand Breaks

**Figure 1:**
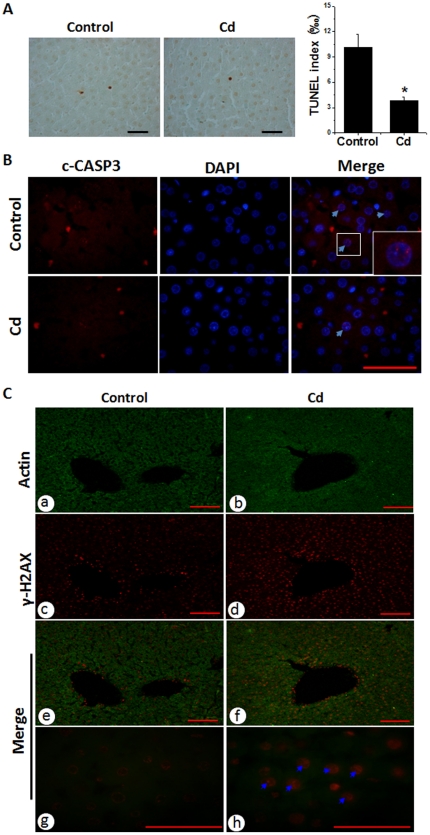
Apoptosis and DNA double-strand break in rat livers. Hepatic tissues were collected from the rats at the 48^th^ week after 4-week exposure to Cd at 20 nmol/kg for TUNEL staining (A, left panel) with a semi-quantitative analysis (A, right panel). Cleaved caspase-3 (c-CASP3) was examined by double immunofluorescent staining for c-CASP3 as red and nuclei by DAPI as blue (B). The sections from these rats were also stained for DNA double-strand breaks (C) with double immunofluorescent stains of β-actin (green, a, b) and γ-H2AX (red, c, d). Bar = 50 µm. Data was presented as mean ± SD. *P<0.05 vs control.

Using immunological examination of the proliferating cell nuclear antigen and Ki67, we have demonstrated the long-lasting increase of cell proliferation in the liver of rats previously exposed to chronic Cd at a low dose [10]. To dissect the mechanism, we further examined the status of hepatic cell death in these rats under the same conditions. TUNEL staining showed a significant low apoptotic cell death in the liver of rats at the 48^th^ week post-exposure, compared to age-matched controls (Figure 1A). Apoptotic cell death was further confirmed by immunofluorescent staining for cleaved caspase-3 (c-CASP3), which shows that livers of rats treated previously with Cd-treated exhibited low levels of nuclear localization of c-CASP3 compared to controls (Figure 1B).

Examination of γ-H2AX as a marker for DNA double strand breaking by dual immunofluorescent staining showed that γ-H2AX expression was significantly increased in the liver of Cd-treated rats compared to age-matched controls (Figure 1C).

### Aberrant Methylation of DNA Promoter CGI Regions in Response to Low-Dose Cd

#### Identification of genomic sites of DNA methylation induced by Cd in rat livers

**Figure 2:**
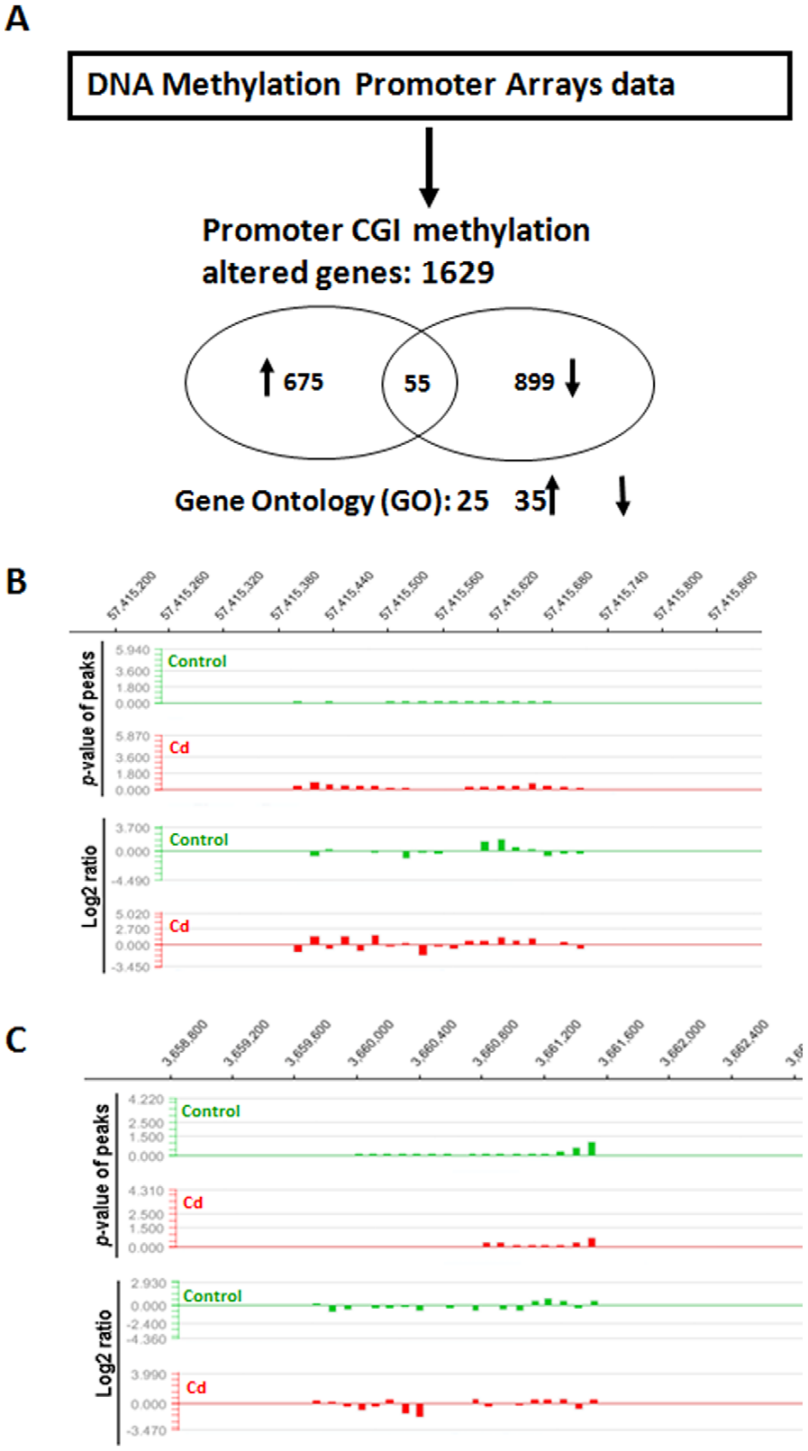
Effects of chronic exposure to Cd to genomic DNA methylation in rat livers. DNA methylation in the livers of rats with and without low-dose Cd treatment was initially profiled with MeDIP-chip, followed by GO analysis as described in *Materials and Methods*. The brief summary of these analyses is outlined (A) with the numbers of total genes with promoter CGI methylation changes (1,629) and the genes, for which all CGIs in the promoter were either hypermethylated (675) or hypomethylated (899). In addition, there were also some genes (55) with mixed hyper- and hypo-methylated CGIs in each promoter. After general analysis for the DNA methylation changes in the livers of rats with and without low-dose Cd treatment as described in A, the methylation profiles of the promoter CGI of CASP8 (B) and TNF (C) genes were selectively analyzed. The NimbleScan software was employed based on the raw data described in Table S1, to generate scaled log_2_-ratio data for IP/input values and *p*-value enrichment data for each probe. Peaks (methylated regions) are then generated based on the *p*-value data. Therefore, the methylation signal levels (Y axis) are represented as either p-value of peaks (top track in each panel) or log_2_-ratio (bottom track in each panel) along with different regions of the promoter gene. For each gene there are only positive numbers relative to base line (0) in term of *p*-value of peaks for each gene, but there are both negative and positive numbers for log_2_-ratio. Conclusion was made by comparing the overall values between control and Cd-treated groups.

To test our hypothesis that chronic exposure to low-dose Cd may cause alteration of DNA methylation in the liver, we initially profiled the DNA methylation status for Cd-treated and age-matched control rats using MeDIP-chip. Genes exhibited either >2-fold change as hypermethylation or <0.5-fold change as hypomethylation in their promoter CGIs caused by Cd are summarized in Table S1. Among the total 1,629 genes with promoter CGI methylation changes, there were 675 genes whose promoter CGIs were hypermethylated, 899 genes whose promoter CGIs were hypomethylated, and 55 genes whose promoter CGIs were mixed with hyper- and hypo-methylation (Figure 2A and Table S1).

#### GO category analysis

GO analysis was applied in order to organize the altered genes found by MeDIP-chip into hierarchical categories (Table S2). In Cd-treated group, genes in 25 GO terms were hypermethylated and genes in 35 GO terms were hypomethylated (Figure 2A). The top 5 significantly up-regulated GO terms include the genes related to receptor regulation, regulation of apoptosis, sensory perception of smell, detection of chemical stimulus involved in sensory perception, and detection of chemical stimulus involved in sensory perception of smell. The top 5 significantly down-regulated GO terms were genes related to MHC class II protein complex, antigen processing and presentation of exogenous peptide antigen, MHC protein complex, antigen processing and presentation of exogenous antigen, and MHC class I protein complex. The GO category analysis results are summarized in Table S3.

#### Promoter CGI methylation profiles of CASP8 and TNF genes

Since the liver of Cd-treated rats showed low incidence of apoptosis (Figure 1), we have focused on the methylation status of genes involved in apoptosis. Promoter CGIs of CASP8 that plays important role in regulation of apoptotic cell death were hypermethylated while promoter CGIs of TNF that is an initiator for death-receptor-dependent apoptosis were hypomethylated (Table S3).

Promoter CGI methylation profiles for CASP8 and TNF genes are shown in Figure 2B and Figure 2C. From the raw data, the NimbleScan software generates scaled log_2_-ratio data for IP/input values and *p*-value enrichment data for each probe. Based on the *p*-value data, we can obtain the *p*-value of peaks (methylated regions), which along with the log_2_-ratio for CASP8 and TNF genes are presented in Figure 2B and Figure 2C, respectively. In terms of *p*-value of peaks for CAPS8, the methylation signals distributed in the range of 57,415,380–57,415,740 regions significantly high relative to the base line (vertical line) in Cd-treated group while these signals in control group was small relative to base line. In terms of Log_2_-ratio for CASP8, methylation signals were shown up and down in different regions relative base line, but the overall signals in Cd-treated groups were high compared to control group (Figure 2B). In contrast, signals in terms of *p*-value of peaks and Log_2_-ratio for TNF are low in Cd group compared to control group (Figure 2C).

**Figure 3:**
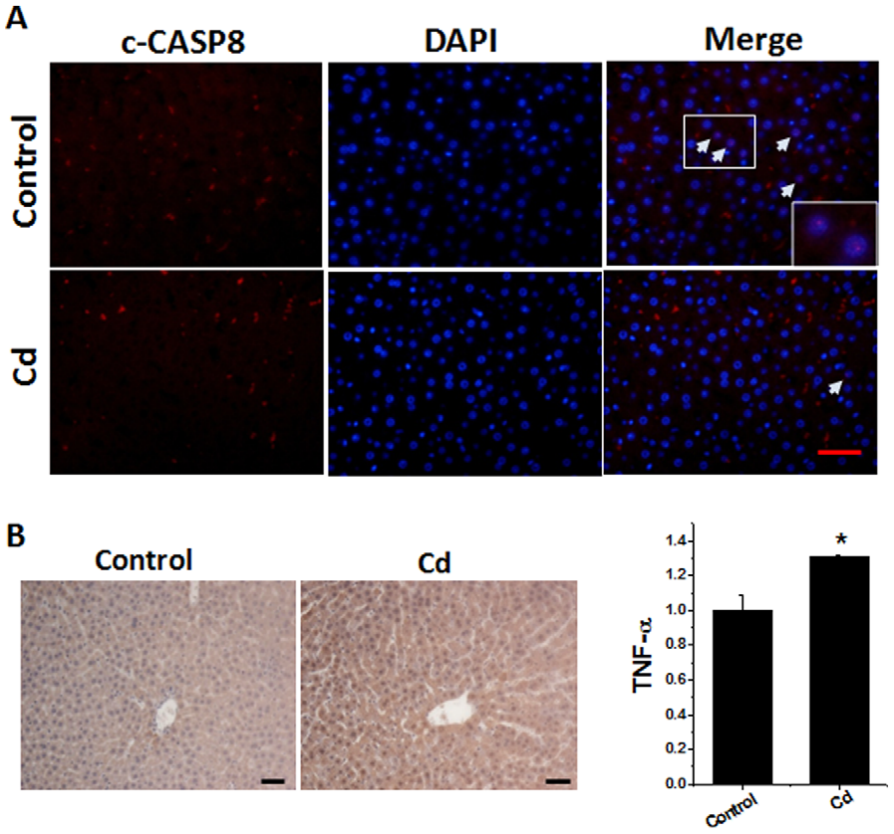
Expression of CASP8 and TNF-α in rat livers. Hepatic tissues were collected from the rats as described in Figure. 1. Cleaved-CASP8 (c-CASP8) was examined by double immunofluorescent staining for c-CASP8 as red and nuclei by DAPI as blue (A). The sections from these rats were also stained for TNF-α by immunohistochemical staining with semi-quantitative analysis (B), as described in Materials and Methods. Bar = 50 µm. Data was presented as mean ± SD. *P<0.05 vs control.

To define whether hypermethylated CASP8 and hypomethylated TNF results in a reduced and increased expression in CASP8 and TNF-α, respectively, in the liver of rats treated previously with low-dose Cd, we immunefluorescently and immunochemically stained CASP8 (Figure 3A) and TNF-α (Figure 3B). Our data clearly revealed that the liver of rats previously treated with low-dose Cd displayed reduced and increased expression of CASP8 and TNF-α, respectively, compared to controls.

### Inhibition of Global DNA Methylation along with Inhibition of CASP8 Promoter Methylation Prevented Cd-Reduced Apoptotic Cell Death

**Figure 4:**
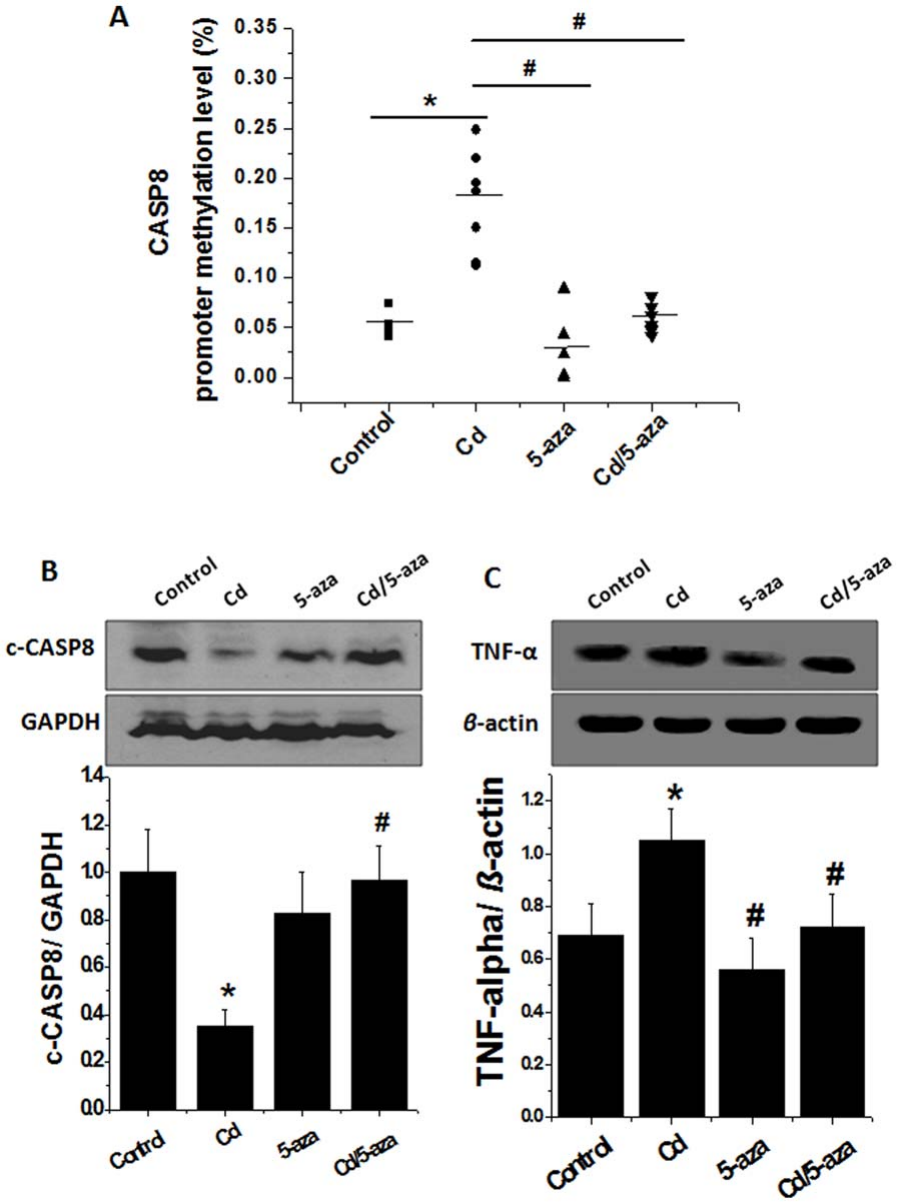
CASP8 gene promoter CGI methylation and TNF-*α* expression in mouse livers. Mice were treated with Cd at 20 nmol/kg for 4 weeks and then were sacrificed at the 56^th^ week after Cd exposure. The Cd-treated and the age-matched mice were given with or without the methylation inhibitor 5-aza for 6 weeks (i.e.: 4 weeks during and 2 weeks after 4-week Cd-treatment). Liver tissues of these four groups of mice were collected for the analysis of CGI methylation status for CASP8 gene promoter with EpiTect Methyl qPCR assay (A), c-CASP8 expression by Western blotting (B), and TNF-*α* expression by Western blot (C). Data was presented as mean ± SD (n = 10). * *p*<0.05 vs control; # *p*<0.05 vs Cd group.

Since CASP8 promoter methylation in the liver of rats exposed to low-dose Cd (Figure 2B) was found to be associated with the significant reduction of apoptotic cell death (Figure 1A), we treated mice that were exposed to same conditions of Cd as used for rats with and without methylation inhibition (5-aza) for 6 weeks (i.e. 4 weeks during and 2 weeks after 4-week Cd exposure) to globally inhibit DNA methylation. The experiment was terminated at the 56^th^ week after 4-week Cd exposure. CASP8 promoter methylation status was examined using real-time PCR, which showed that hepatic CASP8 promoter methylation level was significantly high in the Cd-treat group, but not in the Cd/5-aza-treated group compared to the control (Figure 4A).

Western blotting of CASP8 confirmed that previous exposure to chronic low-dose Cd significantly reduced the cleaved CAPS8 expression in the liver compared to control, the effect of which was completely reversed by 5-aza treatment (Figure 4B).

Since TNF gene was hypomethylated in the liver of rats previously exposed to low-dose Cd (Figure 2C), TNF-*α* protein expression may be increased due to the release of its inhibition by methylation under normal condition as seen by immunological examination in rat livers (Figure 3B). As expected, TNF-*α* protein expression was significantly increased in the liver of Cd-treated mice (Figure 4C). Treatment with 5-aza did not affect hepatic TNF-*α* expression in normal mice, but completely prevented Cd-induced up-regulation of TNF-*α* expression (Figure 4C).

**Figure 5:**
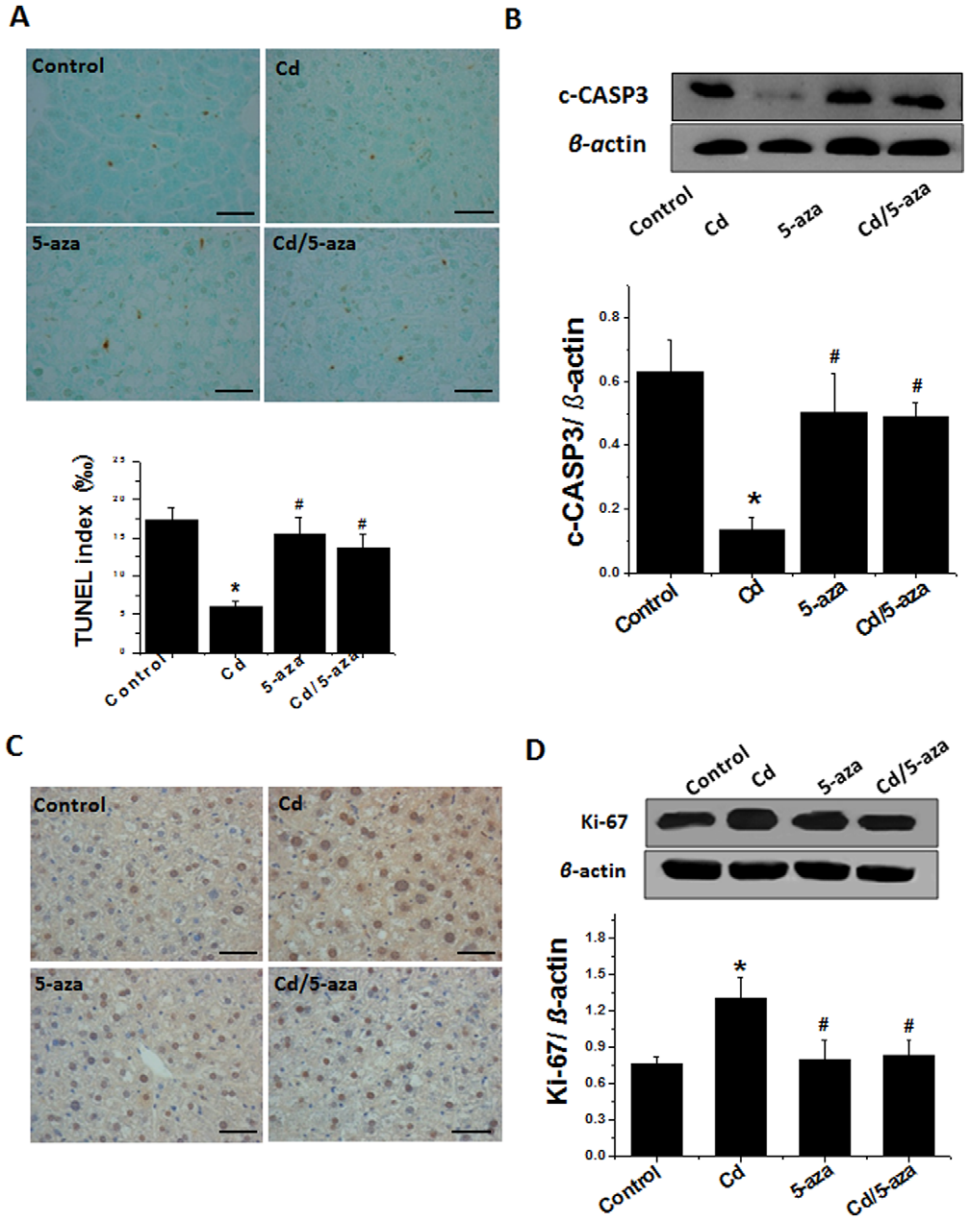
Apoptotic and cell proliferation effects of chronic exposure to Cd on mouse livers. Liver samples were collected as described in Figure 3 for TUNEL staining with semi-quantitative evaluation (A). Apoptotic effect was further confirmed with cleaved-CASP3 (c-CASP3), detected by Western blot (B). Ki-67 measurement with immunohistochemical staining (C) and Western blot (D). The images for each group are the representative ones. Bar = 50 µm. Data was presented as mean ± SD (n = 10). * *p*<0.05 vs control; # *p*<0.05 vs Cd group.

The lack of apoptotic effect of Cd in the liver of rats previously exposed to Cd (Figure 1A) was also observed in the liver of mice previously exposed to Cd by TUNEL staining (Figure 5A). Treatment with 5-aza did not affect the incidence of hepatic apoptotic cell death in normal mice, but restored the apoptotic death level to almost normal level in the Cd/5-aza-treated group. Western blotting of cleaved caspase-3 in liver tissues (Figure 5B) showed that protein expression of cleaved capase-3 was significantly decreased in Cd-treated groups, but not in 5-aza-treated control and Cd/5-aza groups. These results indicate that 5-aza can rescue the apoptotic mechanism that was suppressed by previous Cd-exposure. Therefore, the decrease of caspase-3 cleavage level is associated with reduction of CASP8-dependent cell death due to CASP8 gene CGI hypermethylation and consequently inactivation of CASP8 even though there was an increase in TNF-*α*.

### Inhibition of Global DNA Methylation, including CASP8 Promoter Methylation, Prevented Cd-Increased Steatosis, TGF-β Up-regulation, and Cell Proliferation and Preneoplasia

Hepatic cell proliferation observed in rats previously exposed to chronic Cd [10] was also recaptured in the mouse model by immunohistochemical staining of the cell proliferation marker Ki-67 (Figure 5C). Ki-67 protein expression significantly increased in Cd-group at the 56^th^ week after 4-week exposure to low-dose Cd, while simultaneous treatment with 5-aza for these Cd-exposed mice prevented the stimulating effect of Cd exposure on the cell proliferation. The finding by immunohistochemical assay was confirmed by Western blotting of Ki-67 expression (Figure 5D).

**Figure 6:**
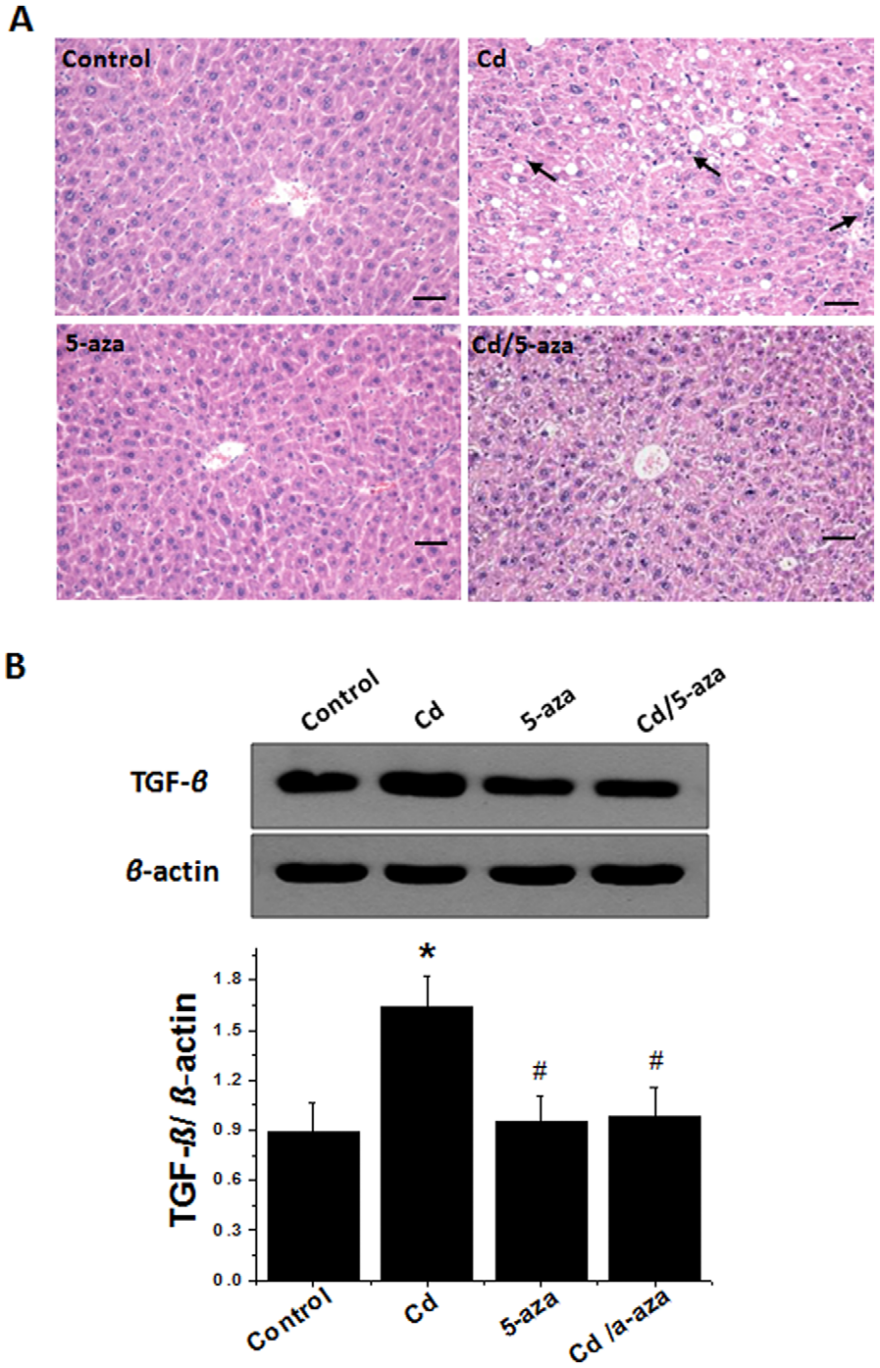
Effects of chronic exposure to Cd on the pathological changes and TGF-*β* expression in mouse livers. Liver tissues collected as described in Figure 4 were used for pathological examination using H&E staining (A), which showed significant increases in lipid drops (vacuoles) with infiltration of inflammatory cells (arrows), and for Western blotting of TGF-β expression (B). Bar = 50 µm. Data was presented as mean ± SD (n = 10). * *p*<0.05 vs control, # *p*<0.05 vs Cd group.

By histopathological examination with H&E staining, livers from Cd-treated group showed the development of steatosis with infiltration of inflammatory cells, an effect that was significantly attenuated by 5-aza treatment in Cd/5-aza group (Figure 6A). Hepatic expression of TGF-*β* was significantly increased in Cd-treated mice compared with controls (Figure 6B). Treatment with 5-aza did not affect TGF-β expression in normal mice, but prevented Cd-increased TGF-β expression.

**Figure 7:**
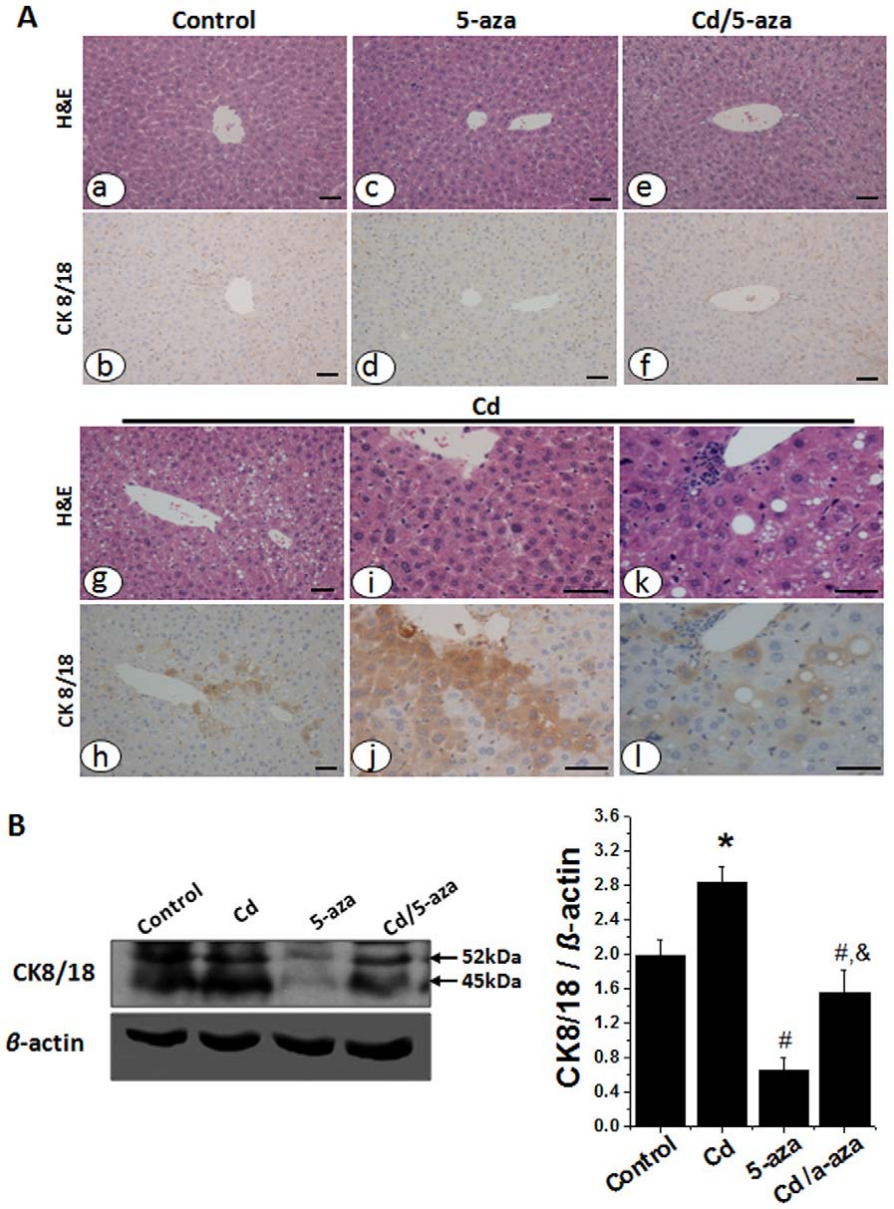
Effects of chronic exposure to Cd on CK 8/18 expression in mouse livers. Expression of hepatic CK8/18 as a new marker of mouse liver preneoplastic change was examined by immunochemical staining (A) and Western blotting (B). In panel A, series of sections of same tissues were conventionally stained with H&E (A-a, c, e, g, i, k) and immunochemically stained with anti-CK8/18 antibodies (A-b, d, f, h, j, l). The results indicated that there was a significant increase in CK8/18 staining foci with little notable change in the same locations using H&E staining. Bar = 50 µm. Data was presented as mean ± SD (n = 10). * *p*<0.05 vs control; # *p*<0.05 vs Cd group; ^&^ *p*<0.05 vs 5-aza group.

As a new marker of mouse liver preneoplasia, CK8/18 over-expression was reportedly found in HCCs and hepatocellular adenomas (HCAs) of B6C3F1 and C57Bl/6J mice treated with chemical carcinogen diethylnitrosamine by immunohistochemistry [11]. To determining if there were liver preneoplastic lesions in the present study, hepatic tissues were stained with the monoclonal anti-mouse CK8/18 antibodies (Figure 7A). There was no significant positive staining in the liver from control, 5-aza and Cd/5-aza groups, but a strong staining of CK8/18 the liver from Cd group, which was further confirmed by Western blotting analysis (Figure 7B).

## Discussion

In our recent study, we found a persistent increase of hepatic cell proliferation in rats at 40^th^ and 48^th^ weeks after 4-week exposure to low-dose Cd [10]#. The present study extends it to show that chronic Cd exposure causes genomic DNA methylation in the liver of rats and mice. Using two animal models that exposed to chronic exposure to low-dose Cd, we demonstrated a causal association of hypermethylation of CASP8 promoter CGIs with the persistently pathological changes in the liver of Cd-exposed rats and mice. We provided the first evidence that hypermethylation of CASP8 gene promoter led to its inactivation along with the loss of death receptor-mediated apoptotic pathway. Therefore, even though there was a significantly increased expression of TNF-*α* in the liver there remained a less apoptotic cell death in the liver of Cd-treated rats and mice.

One of the earliest events in the DNA damage response is phosphorylation of histone H2AX at Ser139 by members of the phosphatidylinositol-3 kinase-like family of kinases to create γ-H2AX; therefore, it has been commonly used to detect DNA damage as a sensitive assay [12]. In the rat model, we demonstrated less hepatic apoptosis but increased DNA double strand breaks, measured by immunofluorescent staining of γ-H_2_AX (Figure 1C) and expression of inflammatory cytokine, TNF-*α* that acts as an important mediator for the receptor-dependent cell death signaling, in the liver of Cd group compared to the age-matched control (Figure 2C and Figure 3B). This suggests that there were persistent toxic effects of previous Cd-exposure in the liver. Consequently, there was a decrease of hepatic apoptotic cell death (Figure 1A,B and 5A,B) that should get rid of the cells with genomic damage and also a persistent increase of hepatic cell proliferation (Figure 5C,D), consistent with a previous finding [13].

In the present study, we used gene microarray chip approach to assess changes in the DNA promoter CGI methylation status in Cd-treated rat livers. There were numerous changes of gene methylation, including up- and down-regulated gene methylation in the liver of Cd-treated rats. DNA methylation is a DNA modification process that controls gene expression. Aberrant DNA methylation has been noticed early during cancer development [14]#. For instance, gene deletion and silence by gene promoter CGI methylation are two major mechanisms of carcinogenicity. Cd has been repeatedly reported to increase DNA methylation [1], [15]. It has been shown that in the cultured cells exposed to 0–1.5 µM Cd, both genomic DNA methylation and enzyme activity of DNA methyltransferase were increased in a concentration-related manner. The cell growth rate was also significantly elevated [16], as reported in other studies [13], [17].

CASP8 is a crucial player in many cell death processes. Silencing of CASP8 gene by hypermethylation of CASP8 promoter CGIs resulted in complete absence of its protein expression, which may explain the absence of apoptotic elimination of the cells with genomic damage and the uncontrolled cell growth; therefore, hypermethylation of CASP8 promoter CGIs has been considered as a common hallmark of relapsed glioblastoma multiforme [18]. CASP8 as a key downstream effector of TNF-*α*-mediated apoptosis has been shown to be frequently hypermethylated in various tumors. Gunawardana *et al*. reported that Cd inhibited both intrinsic and extrinsic apoptotic pathways along with a significant inhibition of CASP8 activity [19]. These separately early observations are supported by our finding of the increase of CASP8 promoter CGI's hypermethylation (Figure 2B) along with a down-regulation of its protein expression (Figure 3A). CASP8 gene promoter CGIs were often methylated in HCC patient's liver tissues, suggesting the potential contribution of CASP8 promoter CGI hypermethylation to HCC probably due to the loss of apoptotic regulation [4].

Although a few studies have shown the induction of apoptotic cell death following exposure to acute Cd alone via different cell death pathways [20], [21], there were other *in vitro* studies to show Cd's inhibition of apoptotic cell death induced by other stressors such as chromium via specific inhibition of caspase-3 activation [22], [23]. These authors assumed that the suppression of apoptosis by Cd may be a significant aspect of Cd carcinogenic mechanism. To establish the direct link of hypermethylated CASP8 gene to the loss of death receptor-mediated apoptotic signaling, we have treated Cd-treated mice with methylation inhibitor 5-aza as used by others [24]#. Treatment with 5-aza restored the apoptotic function in the liver of Cd/5-aza-treated mice (Figure 5A,B).

Another important finding of the present study is the formation of hepatic steatosis along with a significant increase of TGF-β expression in the liver of Cd-treated mice, which was significantly prevented with 5-aza (Figure 6B). It is known that most cases of HCC developed from a pre-existing chronic liver disease, usually due to hepatitis C virus, hepatitis B virus or alcohol. However, world-wide increase in the prevalence (up to 50%) of HCC develops from non-viral-related chronic liver disease, among which non-alcoholic fatty liver disease (NAFLD) has been considered as a predominant risk. Insulin resistance, steatosis, oxidative stress and imbalances in adipokine/cytokine interplay, the most important factors involved in NAFLD pathogenesis and progression, were found to be a determinant role in liver carcinogenesis by promoting cellular growth and DNA damage [25]. Reportedly long-term feeding of mice with either high-fat or choline-deficient diet could induce the formation of hepatic steatosis and inflammation at the early stage, cirrhosis at the middle term (about 60 weeks), and the development of hepatic cancers at the late stage [26], [27], [28]. It is well known that TGF-β plays an important role in the development of hepatic carcinogenesis [29], [30]. It was reported that chronic inflammation associated with hepatitis C virus infection switched TGF-β signaling from tumor-suppression to hepatic fibrogenesis, consequently fibrosis and eventual the development of HCC [29]. In the present study, we demonstrated the information of hepatic steatosis with the infiltration of inflammatory cells in Cd group, but not in others (Figure 6A). Meanwhile, we also showed the upregulation of hepatic TGF-β expression in Cd-treated mice, suggesting the potential development of hepatic fibrosis and eventually cirrhosis, all of which were prevented by inhibition of methylation with 5-aza.

Perhaps one of the most intriguing findings in the present study is the appearance of hepatic preneoplasia in Cd-treated mice (Figure 7) with the newly-established novel marker CK8/18. We applied immunohistochemical staining of CK8/18 for the liver from Cd-treated mice with and without 5-aza, and demonstrated a significant increase in CK8/18 positive cells that are irregularly arranged in the liver only from Cd-treated mice. CK8/18 is a filamentous protein within cells known to influence expression of cell surface receptors, which were recognized by the proteomes of microdissected basophilic foci, HCC and HCA [11]. Significant overexpression of CK8/18 proteins was further confirmed with immunochemical staining in all samples of HCAs and HCCs induced by chemical hepatocarcinogen in B6C3F1 and C57BL/6J mice [11]. To date, this early useful marker has been extensively used in the cancer studies with mouse models [31], [32], [33] and even human tumor tissues [34], [35]. Taken together, these findings of hepatic steatosis formation with inflammation, TGF-β overexpression, imbalance of cell death and proliferation and preneoplastic lesions may suggest the potential risk for the liver, leading to the development of hepatic cancer at the late stage.

In conclusion, findings from the present study indicated that after chronic exposure to low-dose Cd *in vivo*, the liver displayed the aberrant global DNA methylation and a persistent increase in cell proliferation, steatosis and preneoplasia. Thus Cd-induced aberrant DNA methylation could be an early molecular lesion responsible for the inhibition of cell death pathway and stimulation of cell proliferation, along with the formation of steatosis with inflammation and preneoplasia, which may be a possible underlying carcinogenic mechanism of Cd at the late stage.

## Materials and Methods

### Animals and Treatments

Twelve healthy adult male Wistar rats (6-week old) weighing 180–200 g were obtained from the Experimental Animal Center of School of Basic Medical Sciences, Jilin University (Changchun, China). They were housed in plastic cages under standard conditions of light and dark (12 h: 12 h) with an ambient temperature of 24°C±2°C. They were fed with standard laboratory chow and tap water *ad libitum* for 1 week before the experiments commenced. The experiments were approved by and carried out in accordance with the regulation of Animal Experimentation Committee of Jilin University [license No. SYXK(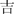)2007–0011 and the animal production license No. SCXK(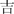)2007–0003].

Since this study was aimed to investigate the late, persistent effects of early (or previous) exposure to Cd at low doses on the liver, i.p. injection of Cd was chosen as the route of administration, instead of dietary administration, to ensure the accurate dose levels and also eliminate absorption influence. Wistar rats were chosen to investigate the effect of Cd on hepatic metal, pathological and total DNA methylation given the reported induction of liver cancer by exposure to Cd despite at a low incidence [36]#. The rats were divided at random into two groups, each comprising six rats; (1) control group: rats were given i.p. injections of physiological saline every other day for 4 weeks. (2) Cd treatment group: rats were given i.p. injections of Cd in saline at 20 nmol/kg every other day for 4 weeks. Cd chloride (CdCl_2_; purity 99.99%) was obtained from Sigma-Aldrich (St. Louis, MO, USA). Although the previous study with Wistar rats employed either single (1–20 µmol/kg) or multiple (5 µmol/kg×4 times) subcutaneous injection of CdCl_2_ to efficiently induce cancer, we attempted to use chronic approach with a lower dosage at 20 nmol/kg daily for 4 weeks, which caused a persistent increase in hepatic cell proliferation until the 48^th^ week after 4-week Cd exposure [10]. Therefore, we also terminated the study at the 48^th^ week after Cd exposure to collect the livers for study. Rats were weighed and sacrificed using pentobarbital sodium (Sigma-Aldrich) at 50 mg/kg body weight, and the livers were removed from animals, rinsed in pre-cooled saline a few times, and then portioned for storage at −80°C.

The second study was to examine whether inhibition of DNA methylation can affect the hepatic methylation and pathological effects of Cd. Due to the consideration of cost for animal maintenance (four groups) and the DNA methylation inhibitor 5-aza-2′-deoxyctidene (5-aza) mouse model was chosen for this part of the study. Like Wistar rats, C57BL/6 mice are relatively resistant to developing spontaneous liver cancer 26,27,28. Although there was no report for the induction of liver cancer directly by Cd in C57BL/6 mice, C57BL/6 mice are considered highly susceptible to Cd-induced hepatotoxicity as the C3H/He and BALB/c mice [37]#. In addition, the following factors were also considered. (1) C57BL/6 mice are highly susceptible to induction of liver cancer by high-fat and choline-deficiency diets [26], [27], [28]; (2) Partial hepatectomy can act as a promoter of hepatic carcinogenesis in C57BL/6J male mice but not C3H/HeJ male mice [38]#; (3) C57BL/6 mice is the mostly employed as a background for many transgenic mouse models. All these features of C57BL/6 mice will be useful for our future studies.

Forty adult male C57BL/6 mice (6-week old), weighing 25 g, were obtained from the Experimental Animal Center University of Wyoming (Laramie, Wyoming, USA). The experiments were approved by and carried out in accordance with the regulation of Animal Experimentation Committee of University of Wyoming (license No. A-3216-01). The mice were randomly divided into four groups (n = 10): (1) control group: mice were given i.p. injections of saline every other day for 4 weeks; (2) Cd treatment group: mice were given i.p. injections of Cd in saline at 20 nmol/kg every other day for 4 weeks; (3) 5-aza treatment group: mice were given i.p. injections of 5-aza-2′-deoxyctidene (5-aza) in saline at 0.25 mg/kg twice weekly for 6 weeks. 5-aza was obtained from Sigma-Aldrich; (4) Cd/5-aza group: mice were given both CdCl2 and 5-aza as groups of (1) and (3). The mice were sacrificed at the 56^th^ week after 4-week Cd exposure (i.e.: 60^th^ week of the study) since the early induction of hepatic steatosis and preneoplasia were reported to develop at about 60 weeks of study in C57BL/6 mice, leading to the induction of hepatic cancers at the more advanced stages [26], [27], [28].

### Genomic DNA Isolation

Genomic DNA was isolated from liver tissues of rats and mice using a QIAamp DNA mini kit (Qiagen, Valencia, CA, USA) according to manufacturer's instructions. Samples were homogenized and treated with 200 µg/ml RNase A for 15 min at room temperature and then digested with 1 mg/ml Proteinase K overnight, followed by exhaustive organic extraction and ethanol precipitation.

### Methylated-Cytosine DNA Immunoprecipitation Microarray Chip (MeDIP-Chip) and Its Analysis

According to the requirement by the MeDIP-Chip assay, at least 25 mg of rat liver DNA sample was diluted in TE buffer (10 mM Tris-HCl, pH 7.5, 1 mM EDTA) and sheared to between 200–300 bp fragments using Bioruptor (Diagenode, Belgium). Four mg of each sample was used as INPUT and the rest heated to 95°C for 10 min and immediately placed on ice. Immunoprecipitation (IP) was performed using 2.5 mg of 5-methyl-cytosine antibody per mg of sheared gDNA in IP buffer (20 mM Na-Phosphate, pH 7.0, 1 M NaCl, 2% Triton-X100). After these samples were rotated overnight at 4°C, they were mixed with 10 ml of 50% protein-A Agarose slurry (pre-washed in 0.1% BSA-PBS and equilibrated in IP buffer). Then samples were rotated for further 2.5 h, followed by 3-time wash with IP buffer, elution using 250 ml lysis buffer (1 M Tris-HCl, pH 8.0, 0.5 M EDTA, 10% SDS, 280 mg/ml Proteinase K) and incubation for 2 h at 55°C. MeDIP was purified and precipitated using phenol and chloroform: isoamyl alcohol. Four mg of INPUT and MeDIP for each sample were labeled with Cy^3^ and Cy^5^, respectively and co-hybridized to the Roch-Nimblegen “RN34 ChIP 385K RefSeq promoter” microarray chip (Roch-Nimblegen, WI, USA). This Roch-Nimblegen array chip comprises of 385,000 isothermal probes of between 50–75 mer lengths with median probe spacing of 100 bps. These probes cover all reported RAT Refseq gene promoters (15,398) that ranged from 2250 bp to +500 bp relative to transcription start sites.

There are two types of signal values of methylation microarray: one is Enrichment, which is the signal value of methylated cytosine 5 specific antibody binding sequence; the other is Input, which represents the signal value of unspecific antibody binding sequence. These two types of signal values could be compared using the MEDME algorithm [39]. The comparison results that are >2 folds or <0.5 fold are deemed as differential methylation. Differential methylation could be classified as absolute methylation (AMS) and relative methylation (RMS). AMS means the methylation level represented by the probes within the range of chromosome regions, whereas RMS means the methylation level of the probes at the same amount of CGI. Signal smoothing treatment is subsequently done for the differentially methylated sites, if differential methylation is found in three or more consecutive probe regions and the tendency of differential methylation is similar, then it could be deduced that differential methylation might be present in the corresponding DNA fragment of the consecutive probes. In the subsequent analysis, we used and showed the AMS results. All data is MIAME compliant and that the raw data has been deposited in a MIAME compliant database (E.g. ArrayExpress, GEO), as detailed on the MGED Society website http://www.mged.org/Workgroups/MIAME/miame.html.

The altered genes defined by the analysis mentioned above were further analyzed using Gene Ontology (GO) database for gene categories (http://www.geneontology.org). Two-side Fisher's exact test and chi-squared test were used for the GO category analysis, and the false discovery rate (FDR) was calculated to correct the *p*-value. *P*-value<0.001 was used as a threshold to select significant GO categories.

### Methylation-Specific Polymerase Chain Reaction

For methylation analysis of mouse liver tissue, the EpiTect Methyl qPCR Assay System (SABiosciences, Frederick, MD) was employed. EpiTect Methyl qPCR Assay is a simple and reliable method for quick detection of the DNA methylation status of the CGI associated with individual gene. This system is capable of detecting DNA methylation with predesigned primers but without bisulfite conversion. DNA methylation-sensitive restriction enzymes and/or methylation-dependent restriction enzymes were used to obtain the products containing hypermethylated DNA sequences or unmethylated DNA sequences, respectively. We digested 125 ng of DNA from mouse liver tissues with Methyl-Profiler™ DNA Methylation Enzyme Kit (SABiosciences) at 37°C for overnight. Then the enzyme was inactivated at 65°C for 20 min. The remaining DNA after digestion is quantified by real-time PCR using primers that flank the region of interests. The CASP8 promoter CGI products were amplified using Real-Time PCR machine (ABI7300, Applied Biosystems, Carlsbad, CA, USA) and were analyzed with the *Ct* values. Used the SABiosciences company's Excel-Based Data Analysis Template, we obtained the percent of hypermethylated DNA. The relative concentration of differentially methylated DNA (specifically hypermethylated, intermediately methylated, and unmethylated DNA) were determined by comparing the amount in each digest with that of a mock digest. The PCR cycling conditions were as follows: 1 cycle at 95°C for 10 min, 40 cycles including 15 s at 97°C and 1 min at 72°C. The PCR product was marked with SYBR® Green. The results obtained according to formula provided by the company.

### Immunohistochemistry for Preneoplasia in Mouse Liver and Other Proteins

Recently overexpression of cytokeratin 8/18 (CK8/18) in the mouse liver has been established as a novel reliable marker of preneoplasia [11], which was also proved and accepted by subsequent several studies [32], [33]. Therefore, liver expression of CK8/18 as the index of preneoplastic lesions was examined with immunochemical staining as others.

Serial sections at 4 µm in thickness were processed for immunolocalization. Briefly, the sections were de-waxed in xylene and rehydrated through descending concentrations of ethanol and the endogenous peroxidases were blocked with 3% H_2_O_2_/methanol for 15 min at 37°C. Sections were pre-incubated in a moist chamber with a protein blocker solution containing 0.1% BSA and 2% non-immune rabbit serum to minimize non-specific staining. Sections were incubated for 1 h at 37°C with the primary antibody diluted in an antibody diluting buffer (anti-ki-67 antibody at 1∶200; anti-CK 8/18 antibodies at 1∶100, both from Enzo Life Sciences, NY, USA; anti-TNF-α antibody at 1∶50 from Abcam, MA, USA), and then with secondary antibody (goat anti-rabbit IgG 1∶300 and goat anti-mouse IgG 1∶300, DAKO ChemMate™, Japan) according to the manufacturer's recommendation. Peroxidases were observed with DAB at room temperature as the chromogen and the sections were briefly counterstained with hematoxylin. Negative controls were prepared by using 0.1% BSA in PBS and non-immune rabbit serum instead of the primary antibody.

### Western Blot Analysis

Forty µg hepatic proteins mixed with SDS-PAGE loading buffer was loaded on 12% SDS-PAGE gel for electrophoresis. Separated proteins were then transferred to nitrocellulose membranes (Axygen, CA, USA). The membrane was first incubated with blocking buffer containing 5% defatted milk, and then incubated with the primary antibodies (anti-Caspase3 at 1∶500; anti-CASP8 at 1∶500; anti-TNF-*α* at 1∶500; anti-TGF-β at 1∶500, all from Cell Signaling, Cambridge, MA, USA; anti-Ki-67 at 1∶1000, Abcam, Cambridge, MA, USA; anti-CK8/18 at 1∶500) for 1 h at 37°C. The membrane was incubated with corresponding secondary antibodies for 1 h at room temperature. Antigen–antibody complexes were visualized with an ECL kit (Thermo Scientific, IL, USA). β-actin was used as the internal control.

### Double Immunofluorescent Staining

For detection of γ-H2AX in the rat liver, immunofluorescent double-staining was used. Sections on Poly-L-lysine solution charged slides were deparaffinized and the antigens were unmasked in a Target Retrieval Solution for 10 min at 98°C and then blocked by 5% normal donkey serum for 20 min and incubated in specific primary antibody combination of both actin and γ-H2AX at 4°C overnight. Primary antibodies used included mouse polyclonal actin antibody (1∶100; Santa Cruz Biotechnology, Inc.), rabbit polyclonal anti-γ-H2AX (1∶100; Abcam), rabbit polyclonal anti-CASP3 (1∶2000; Cell Signaling), and rabbit polyclonal anti-CASP8 (1∶500; Cell Signaling). Sections were incubated with the mixture of two fluorescent conjugated secondary antibodies (FITC conjugated rabbit polyclonal anti-mouse and Cy^3^ conjugated Donkey anti-rabbit) in PBS for 1 h at room temperature. Slides were counterstained with 4′, 6-Diamidino-2-phenylindoldihydrochlorid (DAPI, Sigma-Aldrich) and covered with aqueous mounting medium and analyzed under fluorescent microscope (Nikon, Japan).

### Terminal Deoxynucleotidyl Transferase dUTP Nick-End Labeling Staining of Apoptotic Cells

Sections of the liver from rats and mice were dewaxed, hydrated and subjected to terminal deoxynucleotidyl transferase dUTP nick-end labeling (TUNEL) staining following the instructions provided in the kit (Promega, Madison, WI, USA). The numbers of TUNEL-positive nuclei among the total nuclei were counted under light microscope at magnification 400×. Results were expressed as TUNEL-positive cells/1000 cells.

### Statistical Analysis

Data are expressed as mean ± SD. Statistical methods for DNA methylation analysis has been mentioned in the above section. Other data analysis was done with one-way ANOVA first to determine if differences exist and if so, a Tukey's post hoc test was then used to analyze for the difference between groups with Origin 7.5 laboratory software. Statistical significance was considered as *p*<0.05.

## Supporting Information

Table S1 **Differentially gene promoter methylation in Cd-treated rat livers.**
(XLS)

Table S2 **Summary of GO terms with aberrant gene methylation in Cd-treated rat livers by GO analysis.**
(DOC)

Table S3 **Enriched Gene ontology category in differentially promoter methylated gene in Cd-treated rat livers.**
(XLS)
