# Supplementary material for: Low-Dose Cd Induces Hepatic Gene Hypermethylation, along with the Persistent Reduction of Cell Death and Increase of Cell Proliferation in Rats and Mice
Source: PLoS One. 2012 Mar 23;7(3):e33853. doi: 10.1371/journal.pone.0033853 (PMC3311546; doi:10.1371/journal.pone.0033853)
Supplement: Table S2 — Summary of GO terms with aberrant gene methylation in Cd-treated rat livers by GO analysis. (DOC) [file pone.0033853.s002.doc]

| **Table S2 Summary of GO terms with aberrant gene methylation in Cd-treated rat livers by GO analysis.** | | | | | | |
| --- | --- | --- | --- | --- | --- | --- |
|  | | | | | | |
| **Category ID** | **Names of GO terms** | ***p-*value** | **FDR** | **enrichment** | **go_diffgene**  **_count** | **go_gene**  **_count** |
| **GO terms of genes with hypermethylation** | | | | | | |
| GO:0030545 | receptor regulator activity | 6.06E-05 | 10.8 | 10.8 | 5 | 14 |
| GO:0042981 | regulation of apoptosis | 8.52E-05 | 1.81 | 1.81 | 45 | 729 |
| GO:0007608 | sensory perception of smell | 2.07E-07 | 1.79 | 1.79 | 79 | 1295 |
| GO:0050907 | detection of chemical stimulus involved in sensory perception | 2.24E-07 | 1.79 | 1.79 | 78 | 1275 |
| GO:0050911 | detection of chemical stimulus involved in sensory perception of smell | 3.33E-07 | 1.78 | 1.78 | 77 | 1265 |
| GO:0043067 | regulation of programmed cell death | 0.0001 | 1.78 | 1.78 | 45 | 738 |
| GO:0010941 | regulation of cell death | 0.0001 | 1.78 | 1.78 | 45 | 741 |
| GO:0009593 | detection of chemical stimulus | 3.50E-07 | 1.77 | 1.77 | 78 | 1289 |
| GO:0050906 | detection of stimulus involved in sensory perception | 4.44E-07 | 1.75 | 1.75 | 78 | 1301 |
| GO:0007606 | sensory perception of chemical stimulus | 4.44E-07 | 1.74 | 1.74 | 80 | 1342 |
| GO:0007600 | sensory perception | 8.81E-08 | 1.73 | 1.73 | 91 | 1540 |
| GO:0004984 | olfactory receptor activity | 1.66E-06 | 1.72 | 1.72 | 77 | 1356 |
| GO:0051606 | detection of stimulus | 1.11E-06 | 1.71 | 1.71 | 79 | 1350 |
| GO:0050890 | Cognition | 1.79E-07 | 1.68 | 1.68 | 95 | 1657 |
| GO:0050877 | neurological system process | 4.23E-08 | 1.64 | 1.64 | 110 | 1959 |
| GO:0003008 | system process | 5.76E-08 | 1.59 | 1.59 | 119 | 2187 |
| GO:0004888 | transmembrane receptor activity | 2.32E-06 | 1.52 | 1.52 | 112 | 2229 |
| GO:0004872 | receptor activity | 9.77E-07 | 1.49 | 1.49 | 129 | 2624 |
| GO:0042221 | sensory perception of chemical stimulus | 6.02E-07 | 1.47 | 1.47 | 135 | 2684 |
| GO:0004871 | signal transducer activity | 6.88E-07 | 1.47 | 1.47 | 139 | 2867 |
| GO:0060089 | molecular transducer activity | 6.88E-07 | 1.47 | 1.47 | 139 | 2867 |
| GO:0004930 | G-protein coupled receptor activity | 5.71E-05 | 1.47 | 1.47 | 95 | 1951 |
| GO:0007166 | cell surface receptor linked signal transduction | 9.07E-05 | 1.35 | 1.35 | 131 | 2845 |
| GO:0032501 | multicellular organismal process | 2.81E-06 | 1.3 | 1.3 | 203 | 4575 |
| GO:0050896 | response to stimulus | 1.50E-05 | 1.3 | 1.3 | 179 | 4019 |
| **GO terms of genes with hypomethylation** | | | | | | |
| GO:0042613 | MHC class II protein complex | 5.24E-06 | 11.07 | 11.07 | 6 | 11 |
| GO:0002478 | antigen processing and presentation of exogenous peptide antigen | 9.57E-07 | 9.11 | 9.11 | 8 | 19 |
| GO:0042611 | MHC protein complex | 6.59E-18 | 8.24 | 8.24 | 26 | 64 |
| GO:0019884 | antigen processing and presentation of exogenous antigen | 6.36E-07 | 8.11 | 8.11 | 9 | 24 |
| GO:0042612 | MHC class I protein complex | 2.38E-13 | 7.66 | 7.66 | 20 | 53 |
| GO:0002504 | antigen processing and presentation of peptide or polysaccharide antigen via MHC class II | 7.60E-05 | 7.64 | 7.64 | 6 | 17 |
| GO:0048002 | antigen processing and presentation of peptide antigen | 3.61E-10 | 7.55 | 7.55 | 15 | 43 |
| GO:0002474 | antigen processing and presentation of peptide antigen via MHC class I | 2.85E-06 | 6.95 | 6.95 | 9 | 28 |
| GO:0019882 | antigen processing and presentation | 2.39E-17 | 6.69 | 6.69 | 30 | 97 |
| GO:0006955 | immune response | 2.81E-11 | 2.5 | 2.5 | 61 | 528 |
| GO:0002376 | immune system process | 3.12E-10 | 2.04 | 2.04 | 82 | 868 |
| GO:0004984 | olfactory receptor activity | 2.04E-10 | 1.85 | 1.85 | 107 | 1356 |
| GO:0050911 | detection of chemical stimulus involved in sensory perception of smell | 1.42E-09 | 1.8 | 1.8 | 105 | 1265 |
| GO:0050907 | detection of chemical stimulus involved in sensory perception | 2.20E-09 | 1.78 | 1.78 | 105 | 1275 |
| GO:0007608 | sensory perception of smell | 2.54E-09 | 1.77 | 1.77 | 106 | 1295 |
| GO:0009593 | detection of chemical stimulus | 4.01E-09 | 1.76 | 1.76 | 105 | 1289 |
| GO:0050906 | detection of stimulus involved in sensory perception | 6.63E-09 | 1.75 | 1.75 | 105 | 1301 |
| GO:0007606 | sensory perception of chemical stimulus | 8.96E-09 | 1.73 | 1.73 | 107 | 1342 |
| GO:0051606 | detection of stimulus | 1.24E-08 | 1.71 | 1.71 | 107 | 1350 |
| GO:0007600 | sensory perception | 6.89E-09 | 1.67 | 1.67 | 119 | 1540 |
| GO:0050890 | Cognition | 5.88E-09 | 1.65 | 1.65 | 126 | 1657 |
| GO:0050877 | neurological system process | 1.16E-07 | 1.52 | 1.52 | 138 | 1959 |
| GO:0004930 | G-protein coupled receptor activity | 7.90E-07 | 1.52 | 1.51 | 126 | 1951 |
| GO:0050896 | response to stimulus | 3.67E-15 | 1.49 | 1.49 | 276 | 4019 |
| GO:0003008 | system process | 7.15E-07 | 1.45 | 1.45 | 147 | 2187 |
| GO:0004888 | transmembrane receptor activity | 3.61E-06 | 1.44 | 1.44 | 137 | 2229 |
| GO:0005576 | extracellular region | 0.0002 | 1.44 | 1.44 | 89 | 1253 |
| GO:0042221 | response to chemical stimulus | 7.09E-08 | 1.43 | 1.43 | 178 | 2684 |
| GO:0004872 | receptor activity | 2.75E-06 | 1.4 | 1.4 | 157 | 2624 |
| GO:0004871 | signal transducer activity | 6.05E-06 | 1.37 | 1.37 | 167 | 2867 |
| GO:0060089 | molecular transducer activity | 6.05E-06 | 1.37 | 1.37 | 167 | 2867 |
| GO:0016021 | integral to membrane | 1.50E-05 | 1.24 | 1.24 | 262 | 4292 |
| GO:0031224 | intrinsic to membrane | 2.46E-05 | 1.23 | 1.23 | 267 | 4415 |
| GO:0032501 | multicellular organismal process | 8.35E-05 | 1.21 | 1.21 | 256 | 4575 |
| GO:0044425 | membrane part | 0.0002 | 1.17 | 1.17 | 306 | 5316 |
